# Reply to Giansanti, D. Comment on “Patel, B.; Makaryus, A.N. Artificial Intelligence Advances in the World of Cardiovascular Imaging. *Healthcare* 2022, *10*, 154”

**DOI:** 10.3390/healthcare10040735

**Published:** 2022-04-15

**Authors:** Bhakti Patel, Amgad N. Makaryus

**Affiliations:** 1 Donald and Barbara Zucker School of Medicine at Hofstra/Northwell, Hofstra University, Hempstead, NY 11549, USA; bpatel10@pride.hofstra.edu; 2 Department of Cardiology, Nassau University Medical Center, East Meadow, NY 11554, USA; 3 Department of Cardiology, Northwell Health, Manhasset, NY 11030, USA

Thank you for your interest and comment [1] on our “Artificial Intelligence Advances in the World of Cardiovascular Imaging” publication in *Healthcare* [2] in your Special Issue on artificial intelligence (AI) in Digital Pathology and Digital Radiology (DR) [3]. Our paper focused on documenting information regarding the applications and use of AI in cardiovascular imaging modalities. In reference to your comment, we believe that among the future work in the integration activities of AI in cardiology, there will be acceptance and consensus initiatives based on target investigations and evaluations from users of the technology and beneficiaries (patients) of the technology’s application. Additionally, similar to what has been seen in digital radiology, these initiatives will likely be conducted through questionnaires [4]. Questionnaires seem to be the standard for gathering information regarding patients’ experience and thoughts on the application of artificial intelligence in medicine. A study by Lennartz et al., conducted research where surveys were given ascertaining whether patients preferred a physician or AI’s role in performing a clinical task. The results showed that patients preferred physicians in most clinical tasks or a physician overseeing AI applications [5]. In another study by Ongena et al., the patient acceptance of artificial intelligence was assessed through questionnaire responses. It was found that, overall, patients were distrustful of AI and preferred personal interaction and connections [6]. This information from these example surveys is useful in terms of AI applications and expansion for the future, and how this information should be integrated into the field. These publications also show the significance of questionnaires on providing feedback for the use of AI applications in imaging modalities. Learning from the patient viewpoint can bring a different perspective to help improve the technology to be implemented for the best impact. In the future, we believe with more implementation and usage of AI, patients will be more exposed to the benefits of AI that help improve imaging modalities, and perspectives may change towards patient preference for more AI integration. For that reason, the questionnaires are a great way to survey for acceptance and consensus and should be given regularly as more AI is introduced into the field of medicine in general and cardiovascular imaging specifically. We look forward to new advances and insights to the further application of AI in the world of cardiovascular imaging.

## Data Availability

Not applicable.
